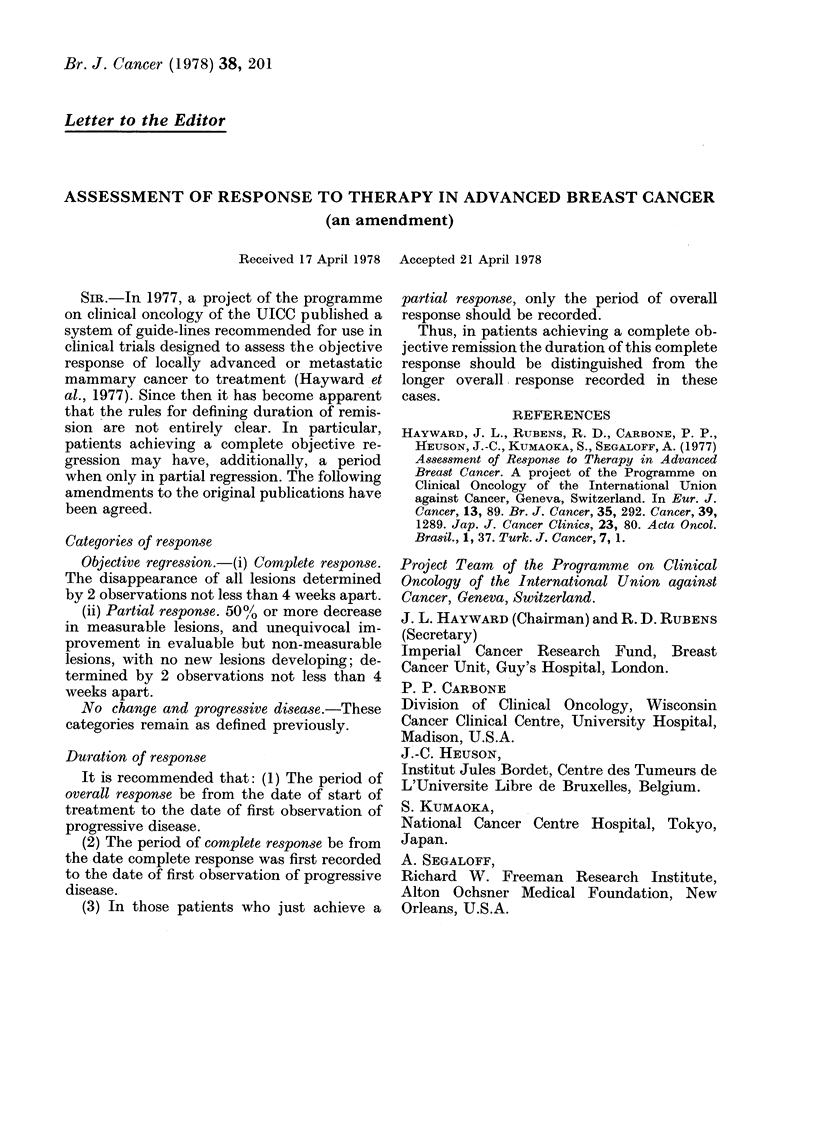# Assessment of response to therapy in advanced breast cancer (an amendment)

**DOI:** 10.1038/bjc.1978.182

**Published:** 1978-07

**Authors:** J. L. Hayward, P. P. Carbone, R. D. Rubens, J. C. Heuson, S. Kumaoka, A. Segaloff


*Br. J. Cancer* (1978) **38**, 201

### *Letter to the Editor*

### ASSESSMENT OF RESPONSE TO THERAPY IN ADVANCED BREAST CANCER (an amendment)

Received 17 April 1978 Accepted 21 April 1978

SIR.—In 1977, a project of the programme on clinical oncology of the UICC published a system of guide-lines recommended for use in clinical trials designed to assess the objective response of locally advanced or metastatic mammary cancer to treatment (Hayward *et al.*, 1977). Since then it has become apparent that the rules for defining duration of remission are not entirely clear. In particular, patients achieving a complete objective regression may have, additionally, a period when only in partial regression. The following amendments to the original publications have been agreed.

*Categories of response*

*Objective regression.*—(i) *Complete response.* The disappearance of all lesions determined by 2 observations not less than 4 weeks apart.

(ii) *Partial response.* 50% or more decrease in measurable lesions, and unequivocal improvement in evaluable but non-measurable lesions, with no new lesions developing; determined by 2 observations not less than 4 weeks apart.

*No change and progressive disease.*—These categories remain as defined previously.

*Duration of response*

It is recommended that: (1) The period of *overall response* be from the date of start of treatment to the date of first observation of progressive disease.

(2) The period of *complete response* be from the date complete response was first recorded to the date of first observation of progressive disease.

(3) In those patients who just achieve a *partial response*, only the period of overall response should be recorded.

Thus, in patients achieving a complete objective remission the duration of this complete response should be distinguished from the longer overall response recorded in these cases.

REFERENCES

HAYWARD, J. L., RUBENS, R. D., CARBONE, P. P., HEUSON, J.-C., KUMAOKA, S., SEGALOFF, A. (1977) *Assessment of Response to Therapy in Advanced Breast Cancer.* A project of the Programme on Clinical Oncology of the International Union against Cancer, Geneva, Switzerland. In *Eur. J. Cancer,* **13,** 89. *Br. J. Cancer,* **35,** 292. *Cancer,* **39,** 1289. *Jap. J. Cancer Clinics,* **23,** 80. *Acta Oncol. Brasil.,* **1,** 37. *Turk. J. Cancer,* **7,** 1.

*Project Team of the Programme on Clinical Oncology of the International Union against Cancer, Geneva, Switzerland.*

J. L. HAYWARD (Chairman) and R. D. RUBENS (Secretary)
Imperial Cancer Research Fund, Breast Cancer Unit, Guy's Hospital, London.

P. P. CARBONE
Division of Clinical Oncology, Wisconsin Cancer Clinical Centre, University Hospital, Madison, U.S.A.

J.-C. HEUSON,
Institut Jules Bordet, Centre des Tumeurs de L'Universite Libre de Bruxelles, Belgium.

S. KUMAOKA,
National Cancer Centre Hospital, Tokyo, Japan.

A. SEGALOFF,
Richard W. Freeman Research Institute, Alton Ochsner Medical Foundation, New Orleans, U.S.A.